# Profile and outcomes of patients admitted with hyperglycemic emergencies in the Buea Regional Hospital in Cameroon

**DOI:** 10.11604/pamj.2021.39.274.14371

**Published:** 2021-08-27

**Authors:** Clovis Nkoke, Luchuo Engelbert Bain, Christelle Makoge, Denis Teuwafeu, Alice Mapina, Cyrille Nkouonlack, Wilfred Kouam, Ahmadou Musa Jingi, Simeon Pierre Choukem

**Affiliations:** 1 Buea Regional Hospital and Clinical Research Education, Networking and Consultancy, Buea, Cameroon,; 2 Clinical Research Education, Networking and Consultancy (CRENC), Douala, Cameroon,; 3 Athena Institute for Research on Innovation and Communication in Health and Life Sciences, Vrije Universiteit, Amsterdam, The Netherlands,; 4 Faculty of Medicine and Biomedical Sciences, University of Yaoundé I, Yaoundé, Cameroon,; 5 Douala General Hospital, Douala, Cameroon

**Keywords:** Hyperglycemic emergencies, diabetic ketoacidosis, hyperosmolar hyperglycemic state, predictors, outcome, Cameroon

## Abstract

**Introduction:**

hyperglycemic emergencies (diabetic ketoacidosis and hyperglycemic hyperosmolar state) are the most common serious acute metabolic complications of diabetes which result in significant morbidity and mortality. There is paucity of data on hyperglycemic emergencies in Cameroon. The objective of this study was to investigate the precipitants and outcomes of patients admitted for hyperglycemic emergencies in the Buea Regional Hospital in the South West Region of Cameroon.

**Methods:**

in this retrospective study the medical records of patients admitted for hyperglycemic emergencies between 2013 and 2016 in the medical unit of the Buea Regional Hospital were reviewed. We extracted data on demographic characteristics, admission clinical characteristics, precipitants, and treatment outcomes. Logistic regression was used to determine predictors of mortality.

**Results:**

data were available for 60 patients (51.7% females) admitted for hyperglycemic emergencies. The mean age was 55.2±16.3 (range 18-86). Overall there were 51 (85%) cases of hyperosmolar hyperglycemic state. Twenty six (43.3%) of the patients had hypertension. The most common precipitants of hyperglycemic emergencies were infections (41.7%), newly diagnosed diabetes (33.3%) and non-adherence to medications (33.3%). Mean admission blood glucose was 574mg/dl±70.0mg/dl. The median length of hospital stay was 6 days. Overall case fatality rate was 21.7%. Six (46.2%) deaths were related to infections. Predictors of mortality were a Glasgow coma score <13(p<0.001), a diastolic blood pressure <60 mmHg (p=0.034) and a heart rate >90(0.057) on admission.

**Conclusion:**

admission for hyperglycemic emergencies in this semi-urban hospital is associated with abnormally high case fatality. Infections, newly diagnosed diabetes and non-adherence to medications are the commonest precipitants of hyperglycemic emergencies. Public health measures to reduce morbidity and mortality from hyperglycemic crisis are urgently needed.

## Introduction

Sub-Saharan Africa is undergoing rapid epidemiological transition with an increase in the prevalence of non-communicable diseases including diabetes [1]. The prevalence of diabetes is 5.7% in urban Cameroon, with an estimated 1 million people living with the disease, 70% of whom remain undiagnosed [2]. Although there have been significant advances in the overall management of diabetes over the last few decades, acute metabolic complications represent a threat to the lives of people living with diabetes. Diabetic ketoacidosis (DKA) and hyperosmolar hyperglycemic state (HHS) are the two most serious acute metabolic complications of diabetes. They are associated with significant morbidity and mortality. The reported mortality rate ranges from 2-5% for DKA and up to 15% for HHS [3, 4]. Few studies have described the clinical features and treatment outcomes of patients with hyperglycemic emergencies in rural settings in sub-Saharan Africa. The aim of our study was to investigate the precipitants and the outcomes of patients admitted for hyperglycemic emergencies over a period of 4 years, in the medical unit of the Buea Regional Hospital.

## Methods

**Study setting:** the study took place in the medical unit of the Buea Regional Hospital. It is a referral hospital in the South West Region of Cameroon. The main economic activity in the region is agriculture. The medical unit of the hospital has a capacity of 50 beds. The diabetes clinic at the hospital provides once weekly outpatient care. Patients admitted to the unit were referred from the emergency department, other departments, out-patients clinics and other hospitals in the region. For each patient, upon discharge or in the event of in-hospital death, both the initial diagnosis and the final diagnosis were recorded in the registers. Recorded diagnoses were usually expanded to include serious co-morbidities. The study was approved by the administrative authorities of the hospital.

**Recruitment and data collection:** the medical records of adult diabetic patients aged ≥18 years with hyperglycemic emergencies admitted between January 2013 and December 2016 were reviewed. For each eligible patient, we extracted data on the socio-demographic characteristics, type of diabetes and duration of treatment, clinical characteristics including admission blood pressure, Glasgow coma score, co-morbidities, and precipitants, laboratory investigations on admission (blood glucose, serum electrolytes, blood urea nitrogen, and serum creatinine), and treatment outcomes.

**Definitions:** diabetic ketoacidosis (DKA) was defined as admission blood glucose >250 mg/l and urine dipstick ketone level ≥ +2. Hyperosmolar hyperglycemic state (HHS) was defined by the presence of significant hyperglycemia (plasma glucose>600mg/dl), alteration in mental status and mild or absent ketonuria.

**Outcome:** the main outcome of the study was in hospital mortality due to hyperglycemic emergencies.

**Statistical analysis:** data collected were analyzed using SPSS version 20. Results are presented as counts and percentages, mean and standard deviation. Student´s t test was used for comparison of means of continuous variables. To see the association between categorical variables, chi-square tests or Fisher exact tests were used. Variables significantly associated with mortality on bivariate analysis were then analysed using multivariate logistic regression. A p-value <0.05 was considered statistically significant.

**Limitations:** there were notable limitations in the present study. First, given the nature of retrospective study, missing data and misclassification biases could occur. Second, small sample size limited the detection of other factors that might be associated with mortality, such as age and other co-morbidities. Despite these limitations, our study has useful data on hyperglycemic emergencies, which can direct public health measures to reduce morbidity and mortality of acute metabolic complications of diabetes.

## Results

**Baseline characteristics:** during the 4 years study period, 60 patients were admitted and treated for hyperglycemic emergencies. Table 1 shows the baseline characteristics of the patients. There were 31(51.7%) females. The mean age was 55.2±16.3 (range 18-86). The mean ages in males and females were similar (56.6 vs 53.9, p=0.53). The majority of the participants (85%) had type 2 diabetes. Overall, 51(85%) of the patients had HHS. Twenty-six (43.3%) of the patients had hypertension. Three patients (5%) had previous stroke and 1 patient had known coronary artery disease. Forty (66.7%) patients were known to have diabetes, while the remaining 20(33.3%) were newly diagnosed with diabetes mellitus. The mean admission blood glucose was 574 mg/dl±70.0 mg/dl. Table 1 shows a comparison of DKA and HHS. Patients with HHS were significantly older (p<0.001) and also had a significantly higher Glasgow coma score (p=0.006) and diastolic blood pressure (p=0.014). Data are presented as mean ±standard deviation; *p for comparison between DKA and HHS; DKA: diabetic ketoacidosis; GCS: Glasgow coma score; HHS: hyperosmolar hyperglycemic state.

**Table 1:** baseline characteristics of patients with hyperglycemic emergencies

Variable	DKA(n=9)	HHS(n=51)	p value*
Age(years)	32.8±13.1	59.2±13.5	<0.001
Admission blood glucose(mg/dl)	388±90.3	>600	<0.001
SBP(mmHg)	110.2±23.6	133.2±27.6	0.023
DBP(mmHg)	67.2±15.6	82.8±17.3	0.014
Serum potassium(mmol/l)	3.9±0.7	4.3±1.0	0.48
Serum Sodium(mmol/l)	142±15.85	138±7.02	0.48
Serum creatinine(mg/dl)	2.64±1.94	1.53±1.28	0.076
GCS	11.9±4.3	14.2±1.7	0.006

**Figure 1:**
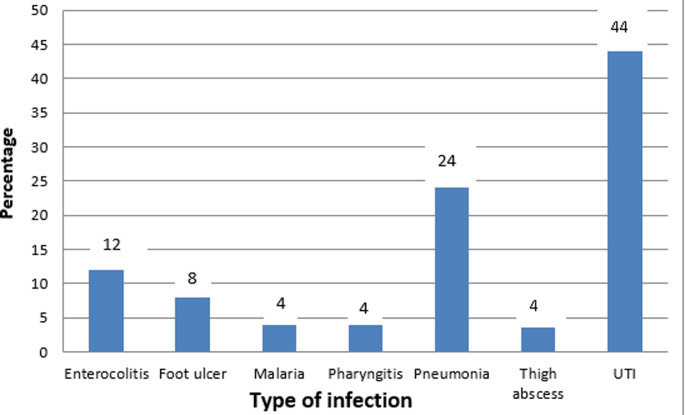
distribution of types of infections

**Precipitating factors of hyperglycemic emergencies:** precipitants of hyperglycemic emergencies were infections in 25 patients (41.7%); newly diagnosed diabetes in 20 patients (33.3%), and non adherence to medications in 20 patients (33.3%). Figure 1 shows the distribution of types of infections.

**Outcome of patients with hyperglycemic emergencies:** the median length of hospital stay was 6 days. The mean duration of hospitalization was shorter in patients who died (3.2 vs 6.2 days; p=0.001). Table 2 shows the predictors of mortality. The overall in-hospital mortality due to hyperglycemic emergencies was 13 (21.7%). Mortality was 2 (28.5%) in DKA and 11 (27.5%) in HHS. Out of the 13 patients who died, 6 (46.2%) had infections. In a multivariable model, predictors of mortality on admission were a GCS<13, and a diastolic blood pressure <60mmHg. Table 2 shows the predictors of in-hospital mortality.

**Table 2:** predictors of mortality

Predictor	aOR (95% CI)	p value
Male sex	1.89 (0.54 - 6.62)	0.315
Age < 40 years	0.4 (0.58 - 2.03)	0.258
Creatinine > 15 mg/l	2.75 (0.33 - 22.9)	0.336
DBP < 60 mmHg	4.89 (1.03 - 23.3)	0.034
DBP > 90 mmHg	1.08 (0.29 - 4.09)	0.915
SBP <100 mmHg	2.58 (0.53 - 12.6)	0.220
SBP > 140 mmHg	0.5 (0.12 - 2.06)	0.332
Heart rate > 90 bpm	16.37 (0018 - 292.1)	0.057
Fever	1.38 (0.39 - 4.91)	0.623
GCS<13	24.8 (5.2-117.8)	<0.001
Infection	1.31 (0.38 - 4.5)	0.669
Newly diagnosed DM	0.81 (0.22 - 3.03)	0.754

## Discussion

In this study, we have reported the profile and outcomes of patients admitted for hyperglycemic emergencies in a semi-urban setting in Cameroon. The results show that HHS was the most common diagnosis among diabetic patients admitted for hyperglycemic emergency, and infection was the most common precipitating factor of hyperglycemic emergencies. In hospital case fatality was high and independent predictors of mortality on admission were a low Glasgow coma score, a low diastolic blood pressure and a high heart rate. There is paucity of data on hyperglycemic emergencies in resource limited settings. We found that 85% of admissions for hyperglycemic emergencies in this semi-urban hospital were due to HHS. In a similar study in Ethiopia, admission for hyperglycemic emergencies was dominated by DKA with a proportion of 93% [5]. This high proportion of DKA could be partly attributed to the fact that about 64% of the patients in their study were patients with type 1 diabetes. In an urban medical setting in Yaoundé, the capital city of Cameroon, the proportion of hyperglycemic emergencies (HHS and DKA) among patients admitted for diabetic coma was 50% [6]. In that study, the proportion of HHS among patients with hyperglycemic emergencies was 50% [6]. The higher proportion of HHS in this study may be due to the fact that the majority of the patients were adult patients with type 2 diabetes.

The most common precipitating factors of hyperglycemic emergencies were infections in 41.7%, newly diagnosed diabetes in 33.3% and non adherence to medications in 33.3%. Like in many other studies, infections have been reported as the most common precipitating factor of hyperglycemic emergencies [5,7-10]. Higher proportions of infection have been reported in Thailand, Nigeria and Ethiopia where they represented 71.4%, 57% and 59% respectively [5,10,11]. These results highlight the importance of infection prevention and drug compliance to prevent hyperglycemic emergencies. The proportion of non-adherence in the present study was comparable to that reported in Ethiopia [5]. The high rates of non-adherence to medications and newly diagnosed diabetes in emergency reflect poverty and low therapeutic education, and raise the need for mass screening as many patients remain undiagnosed of diabetes and present for medical attention after developing diabetic complications and worsening of the conditions. The median length of hospital stay in this study was 6 days. This finding was similar to that reported by Desse *et al*. in Ethiopia but far lower than that reported by Ezeani *et al*. in Nigeria [5,11]. It was however higher than that reported in other studies [12,13]. Patients who died in the present study had a significantly shorter duration of hospital admission compared to patients who did not die suggesting that infections could be an important contributor to precocious death in patients admitted for hyperglycemic emergencies. A possible explanation for a shorter duration of hospital stay in this study may be attributed to the initial patient presentation. It has been reported that diabetic foot ulceration is associated with longer duration of hospital stay [11,13]. But only two patients in this study (8%) had diabetic foot ulcerations.

The overall case fatality in this study was 21.7%. This was comparable to that reported in South Africa, with an overall mortality of 20.2% [14]. On the contrary, other studies in other resource limited settings reported a significantly lower mortality. In Nigeria, Ezeani *et al*. reported a mortality of 4.8% [11]. In Ethiopia, the mortality was 9.8% [5]. In Jamaica, patients admitted for hyperglycemic emergencies also had a lower mortality [15]. In this study, the mortality due to HHS and DKA were comparable. In a study in Thailand, Anthanont *et al*. reported a higher mortality rate for HHS compared to DKA (10). A similar high mortality for HHS was reported in other studies [15,16]. In an urban medical center in the capital city of Cameroon, Dehayem *et al*. reported case fatality rates of 7.7% and 15.4 % among patients with DKA and HHS respectively [6]. This difference in mortality may be due to differences in patient management with no standardized protocols, precipitating factors, age, co-morbidities, poverty in the semi-urban setting and absence of adequate laboratory investigations at presentation, inadequate laboratory monitoring during treatment to monitor patient response. The majority of patients who died in this study had infections (46.2%). Infections are an important cause of death in patients admitted for acute metabolic complications of diabetes. In an urban medical center in Yaoundé, Dehayem *et al*. reported infections to be the ultimate cause of death in more than 50% of those who died during admission for acute metabolic complications of diabetes [6]. This high mortality rate in the present study emphasizes the importance of aggressive treatment of infections and monitoring the process in diabetic patients admitted for hyperglycemic emergencies and other acute metabolic complications.

## Conclusion

This study has shown that infections, non-adherence to treatment and newly diagnosed diabetes were the most common precipitants of hyperglycemic emergencies. Case fatality rate was abnormally high. Public health measures to reduce deaths from hyperglycemic crisis are urgently needed.

### What is known about this topic

- Hyperglycemic emergencies are the most serious acute complications of diabetes mellitus;
- They are associated with high mortality.

### What this study adds

- Admission for hyperglycemic emergencies in the study setting is associated with high mortality;
- Infections were the most common precipitating factors of hyperglycemic emergencies;
- An altered level of consciousness and a low diastolic blood pressure were significant predictors of mortality.
